# Omalizumab effectiveness in patients with a previously failed oral immunotherapy for severe milk allergy

**DOI:** 10.1002/iid3.542

**Published:** 2021-10-08

**Authors:** Laura Badina, Beatrice Belluzzi, Sarah Contorno, Benedetta Bossini, Elisa Benelli, Egidio Barbi, Irene Berti

**Affiliations:** ^1^ Clinica Pediatrica Institute for Maternal and Child Health ‐ “IRCCS Burlo Garofolo” Trieste Italy; ^2^ Department of Medical Sciences University of Trieste Trieste Italy

**Keywords:** anaphylaxis, asthma, avoidance diet, IgE‐mediated food allergies, IgE‐mediated reactions, OIT failure, omalizumab, oral immunotherapy, severe milk allergy

## Abstract

**Background:**

Some studies addressed the issue of omalizumab (OML) effectiveness in children starting their first oral immunotherapy (OIT) attempt but no study investigated the possible role of OML in the setting of patients with persisting milk allergy after a failed OIT attempt.

**Methods:**

Single‐center, prospective, observational study in a selected group of patients with a persisting and severe cow milk (CM) allergy associated with moderate allergic asthma, in which a previous OIT attempt had already failed. We performed an open oral food challenge (OFC) to identify patients who tolerated less than 173 mg of cow's milk protein. At the end of the recruitment, we have found four patients with a mean age of 16.25 years (8–24) who had suspended a previous OIT attempt and still reacted to an amount of CM equal or below 173 mg. Enrolled patients, after an 8‐week course of OML along with a CM avoiding diet, underwent again an open OFC with CM to re‐evaluate their threshold. Eventually, a new OIT course was started using the same OIT protocol of the previous attempt, maintaining cotreatment with OML for the first 12 months. For each patient, we documented: the threshold of CM at OFC, level of specific immunoglobulin E (IgE) and IgG4 for milk, and quality of life (QoL).

**Results:**

During OIT the four patients experienced no reactions or extremely mild ones (oral itching, transient mild abdominal pain). All increased their threshold of CM in OML if compared with the baseline and maintained it long after that biologic therapy had discontinued. Specific milk proteins IgG4 levels significantly increased in all.

**Conclusion:**

In this series, OML was effective in patients with severe CM allergy who had previously failed OIT, allowing milk intake without adverse reactions and improving the QoL.

## 1 MAIN TEXT

Immunoglobulin E (IgE)‐mediated food allergies represent the first cause of anaphylaxis in children. About 4% of children are allergic to at least one food and cow's milk (CM) is the most common food allergen involved.^1^ Cow's milk allergy (CMA) is generally considered a disease with a good prognosis because tolerance is spontaneously acquired over time in most cases. In patients that continue to have allergic reactions after ingestion of traces of CM at age of 5 or more, especially if they have high specific IgE levels for CM and CM proteins, the development of a persistent CMA is more likely. In these cases, the only therapeutic chance is represented by oral immunotherapy (OIT), consisting of ingesting an increasing amount of milk to reduce allergic reactivity and anaphylactic risk.^1^ Unfortunately, this approach carries a risk of systemic adverse reactions resulting in discontinuation of OIT in approximately 20% of patients.^2^, ^3^ Omalizumab (OML), an anti‐IgE monoclonal antibody that binds circulating IgE and reduces IgE‐receptor affinity, has been proposed for reducing allergic reactions rate in course of OIT.^4^ It could be considered for increasing OIT effectiveness and safety, even if, currently, it is approved in Europe only for the treatment of moderate‐severe asthma and chronic spontaneous urticaria.

Some studies addressed the issue of OML effectiveness in children starting their first OIT attempt, showing a better safety record without a significant improvement in the final acquisition tolerance outcome.^4^ No study investigated the possible role of OML in the setting of patients with persisting milk allergy after a failed OIT attempt.

We have conducted a single‐center, prospective, observational study in a selected group of patients with a persisting and severe CMA associated with moderate allergic asthma, in which a previous OIT attempt had already failed. OML therapy was started with a double aim: improve asthma's control, known risk factor for severe anaphylaxis, and perform a second OIT attempt. The study was approved by the Ethical Committee (IRB 05/17). Written informed consent to participate in the study was obtained from parents and patients.

We enrolled 33 patients who failed a previous OIT attempt because of repeated allergic reactions. Among these, we excluded those who did not present moderate or severe asthma as defined in the GINA main report. Out of the selected 20 patients, 9 agreed to the study.

We performed an open oral food challenge (OFC) to identify patients who tolerated less than 173 mg of cow's milk (CM) protein. At the end of the recruitment, we have found four patients with a mean age of 16.25 years (8–24) who had suspended a previous OIT attempt between 1 and 5 years earlier and still reacted to an amount of CM ≤173 mg.

As far as food allergy is concerned all four patients were allergic to CM protein only at the time of enrollment, with one of them with a history of a previous wheat allergy.

All patients were allergic to respiratory allergens as well, with sensitization to at least one perennial allergen such as house dust mites, mold, cat, or dog. Two patients were also allergic to grass pollen.

Enrolled patients, after an 8‐week course of OML along with a CM avoiding diet, underwent again an open OFC with CM to re‐evaluate their threshold.

The OML dose was given according to s‐IgE values and weight, without exceeding the maximum recommended dose (600 mg) for asthma treatment.

Eventually, a new OIT course was started using the same OIT protocol of the previous attempt (as described in Longo et al.^3^), maintaining cotreatment with OML for the first 12 months.

For each patient, we documented: the threshold of CM at OFC, level of specific IgE and IgG4 for milk and its specific proteins (lactalbumin, lactoglobulin, casein), and quality of life (QoL) (Tables 1 and 2), at the beginning of OIT, at the suspension of OML therapy and after 6 months in which OIT has continued without OML. The QoL was evaluated with a specific validated Food Allergy Quality of Life Questionnaires (FAQLQ) for children, Children Form (8–12 years) or for adolescents, Teen Form (>13 years).

**Table 1:** Specific IgE and IgG4 levels for milk and its proteins (lactalbumin, lactoglobulin, and casein) were evaluated at the baseline and after 2 months of the suspension of omalizumab (OML) for all four patients

	Pt 1 at the baseline	Pt 1 after OML	Pt 2 at the baseline	Pt 2 after OML	Pt 3 at the baseline	Pt 3 after OML	Pt 4 at the baseline	Pt 4 after OML
Specific IgE (kU/L)								
Milk	>100	>100	>100	>100	>100	81	46.2	>100
Lactalbumin	20.90	61.70	40.80	>100	68.90	44.50	3.51	17.8
Lactoglobulin	68.90	>100	11.70	>100	80.50	51.10	2.81	14.2
Casein	>100	>100	91.50	>100	>100	81.30	52	100
IgG4 (mgA/L)								
Milk	11.4	204	28	>100	10.6	94.9	8.44	25.4
Lactalbumin	0.41	7.36	6.17	>100	0.45	22.80	0.11	2.95
Lactoglobulin	1.20	35.10	0.33	3.61	0.33	20	0.07	1.29
Casein	5.25	96.40	12.20	>100	1.06	10.40	1.56	16.8

*Note*: As reported in other studies, a transient IgE increment is expected during the OML treatment, but it is not already known how long it could last.

Abbreviation: IgE, immunoglobulin E.

**Table 2:** In order from left to right: the reactivity threshold registered by an oral food challenge (OFC) at the baseline; the reactivity threshold at the OFC after 2 months of the beginning of only omalizumab (OML); starting mg cow's milk protein for OML‐enabled oral immunotherapy (OIT) (maximum tolerated at the OFC); maintaining dose at home during OML‐enabled OIT; the reactivity threshold after 2 months of OML suspension and continuing OIT; the maintaining dose after 6 months of OML suspension and continuing OIT

	Baseline threshold (mg)	Threshold after 2 months of OML (mg)	Starting dose (mg)	Maintaining dose at home (mg)	Reduced dose after OML withdrawal (mg)^a^	Threshold after 2 months of OML stop (mg)	Maintaining dose after 6 months of OML stop (mg)	FAQLQ at the baseline	FAQLQ after 6 months of OML stop
Pt 1	68	204	170	850	510^a^	Unknown^b^	850	160	93
Pt 2	68	340	272	1292	850^a^	Still in OML	Still in OML	164	156
Pt 3	51	272	204	2550	340^a^	476	408	153	140
Pt 4	68	136	68	2040	1428^a^	3060	2720	97	85

*Note*: All data are expressed in mg of cow's milk protein (100 ml of milk = 3.4 g of protein).^6^ Quality of life was evaluated with Food Allergy Quality of Life Questionnaires (FAQLQ): lower scores correspond to a better quality of life.

^a^ These doses were reduced due to prudential safety concerns after the drug's withdrawal and not for symptoms.

^b^ We stopped the OFC at 612 mg of cow's milk protein because of the patient's refusal to continue the test.

At the suspension of OML, considering the theoretical risk of reactions induced by loss of OML protection, we prudentially reduced by 30% the maintenance dose of CM that every patient had achieved. After an event‐free 6‐month interval from OML discontinuation, patients were allowed to slowly increase the milk protein dose and a reassessment of the tolerated dose and related symptoms was performed. During the study period, allergic reactions and OML side effects were recorded.

During OIT patients experienced no reactions or extremely mild ones (oral itching, transient mild abdominal pain) and no adverse effect related to OML was recorded.

All patients increased their threshold of CM in OML if compared with the baseline and maintained it long after that biologic therapy had discontinued. All participants improved their asthma control, reducing the need for as‐needed albuterol and the dose of inhaled steroids. After 2 months of the OML discontinuation, one patient (Patient 2) resumed OML, because of deterioration in control of asthma. Due to a high level of fear and anxiety, one subject (Patient 3) chose to drastically reduce the CM maintenance dose after OML withdrawal even without any reaction. All patients have maintained a clinically meaningful improvement in FAQLQ after OML‐enhanced OIT. As far as FAQLQ is concerned there is no well‐established cut‐off to determine a poor versus a good QoL, but all patients reported to be relieved by their improvement. The QoL typical reported feature specifically concerned the improvement of outdoor life and social life. Reduced anxiety and loss of fear of severe reactions due to food contaminations were the main issues.

This series showed that OML was effective in patients with severe CM allergy who had previously failed OIT, allowing milk intake without adverse reactions and improving the QoL. In such a selected population, the maintenance of the threshold of CM ingested without adverse reactions after OML discontinuation represented a pivotal issue, although follow‐up was still limited to a maximum period of 18 months.

Previous studies reported the effectiveness of OML‐enabled OIT but presented a different set of patients in terms of younger age, lower severity of the allergy, and first OIT experience.^4^, ^5^ It is still not clear if patients in these series would have improved their milk allergy even without OML, but only with OIT. In fact, while these reports have shown that OML has promising results in reducing the adverse effects of OIT, its possible role in selected patients with very severe disease and previous OIT failure has not been investigated so far. To our knowledge, this is the first study to assess a sustained tolerance of milk‐OIT off OML in such a highly selected population with specific inclusion criteria, as asthma and a previous OIT‐failure.

The limits of this report include the small sample size and the follow‐up still in progress.

Due to the limits of this study far more data are needed to establish the safety and effectiveness of this approach, even though we believe that these results are a proof of concept that deserves to be shared for further trials and investigations.

## CONFLICT OF INTERESTS

The authors declare that there are no conflict of interests.

## AUTHOR CONTRIBUTIONS

All authors contributed to the article's conception and design. Sarah Contorno, Beatrice Belluzzi, and Benedetta Bossini wrote the first draft of the manuscript. Sarah Contorno, Elisa Benelli, and Laura Badina conceptualized the study. Laura Badina, Irene Berti, and Egidio Barbi critically reviewed the manuscript. All the authors read and approved the final version of the manuscript as submitted.
